# Supplementary material for: Gender Differences in the Relationship Between Health Literacy and Stress Among Caregivers of Older Adults with Dementia
Source: Healthcare (Basel). 2025 Nov 26;13(23):3064. doi: 10.3390/healthcare13233064 (PMC12691947; doi:10.3390/healthcare13233064)
Supplement: Supplementary file 1 [file healthcare-13-03064-s001.zip › healthcare-3879006-supplementary.pdf]

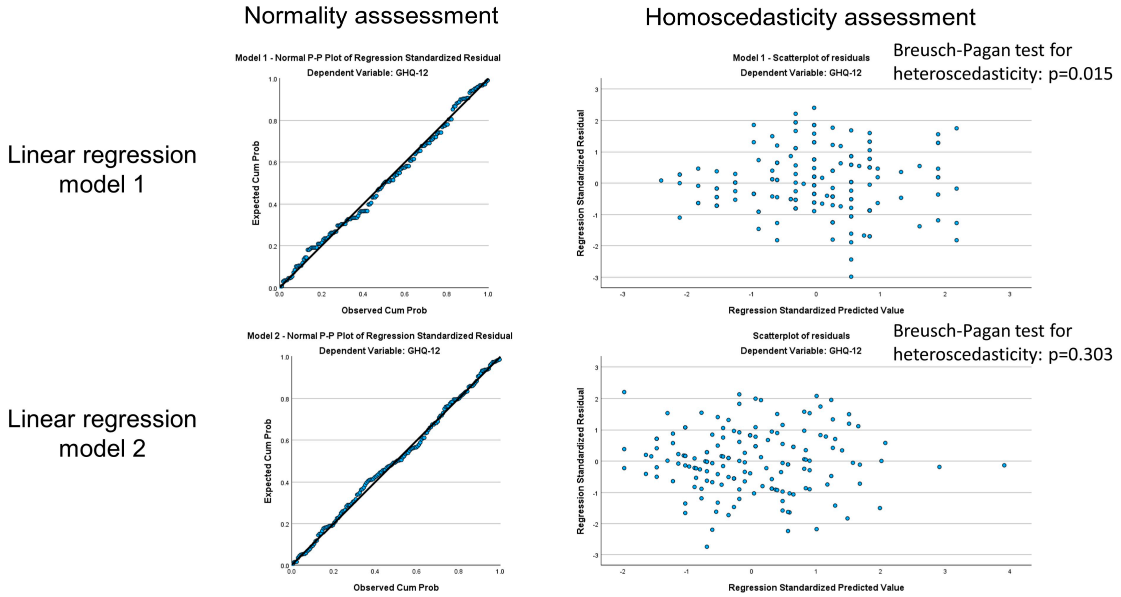

Supplementary Figure S1. Assessment of assumptions for linear regression models 1 and 2 (see Table 4). For each of the models, the normal P-P plot is reported to check for normal distribution of residuals and scatterplot of residuals is reported to check for homoscedasticity (confirmed for model 2).
